# The Administration of Inactivated and Stabilized Whole-Cells of *Saccharomyces cerevisiae* to Gestating Sows Improves Lactation Efficiency and Post-Weaning Antimicrobial Use

**DOI:** 10.3390/vetsci10090576

**Published:** 2023-09-18

**Authors:** Annalisa Scollo, Irene Borello, Marco Ghilardi, Alberto Cavagnini

**Affiliations:** 1 Department of Veterinary Sciences, University of Torino, 10095 Grugliasco, TO, Italy; irene.borello@edu.unito.it; 2 Dox-al Italia S.p.A., 20884 Sulbiate, MB, Italy; marco.ghilardi@doxal.com; 3 Struttura s.r.l., 25025 Manerbio, BS, Italy; alberto.cavagnini@ecopork.it

**Keywords:** lactation efficiency, *Saccharomyces cerevisiae*, antibiotic use, piglets, sow

## Abstract

**Simple Summary:**

Interest towards postbiotics and their beneficial effects on animal health and production is increasing in the era of modern pig farming considering the growth of the cost of raw materials, the demand for increasingly performing animals and, at the same time, the need to de-crease the consumption of antibiotics. The aim of the present study was to investigate the effects on the mother and litter of a postbiotic from inactivated and stabilized whole-cells of *Saccharomyces cerevisiae* administered to the sow during gestation, following piglets up to the post-weaning phase. The results suggest that dietary supplementation of inactivated and stabilized whole-cells of *Saccharomyces cerevisiae* from yeast culture during the gestation of sows can potentially improve lactation efficiency, mortality of the piglets and antibiotic use during the weaning phase. Results related to the lactation efficiency suggest its possible future role in the so-called “cellular agriculture” that should be investigated.

**Abstract:**

Increasingly hyperprolific sows and the need to reduce antibiotics represent challenges in pig farming. The aim of this work was to determine the effects of a postbiotic obtained from inactivated and stabilized whole-cells of *Saccharomyces cerevisiae*, administered during the sow’s gestation, on the performance of the mother and litter. Maternal feed intake, productive parameters, colostrum quality and post-weaning piglets’ health were assessed, including antibiotic consumption. The trial involved 183 sows, divided into two groups: (1) sows fed with a daily supplementation of postbiotic during gestation (*n* = 90); (2) sows without any supplement (*n* = 93). Piglets were followed up at two different post-weaning sites. The lactation efficiency of the treated sows improved by +5.9% (41.3 ± 11.4 vs. 35.4 ± 11.6%; *p* = 0.011). Lactating piglets’ mortality was lower in the treated group (25.1 ± 16.7 vs. 28.8 ± 14.4%; *p* = 0.048). The same tendency was shown in both the weaning sites, together with a reduced antibiotic consumption in weaning site 1 (0.72 ± 0.25 vs. 1.22 ± 0.30 DDDvet/PCU; *p* = 0.047). The results suggest the role of this postbiotic administered to the mother in improving the health status of the piglets. Furthermore, lactation efficiency is suggested as an interesting parameter for assessing the efficiency of farming.

## 1. Introduction

Genetic selection has led to the production of ever more hyperprolific sows, increasing the need for nutrients during gestation and lactation to simultaneously allow for an increase in milk production to support litter growth [1]. Limited nutritional intake can lead sows to a severe catabolic state and, consequently, reduced reproductive performance [2,3]. This is why nutritional strategies to improve milk production and ejection have acquired particular importance in recent times, considering that improving the performance of the litter is also equivalent to greater economic sustainability of the sow farm. Furthermore, the need to wean more “ready and mature” animals from a digestive point of view has also increased in recent years, as the abolition of the use of zinc oxide in the European Union since the mid-2022 and the request to reduce antibiotics in the livestock sector certainly represent challenges in pig farming. Increased prolificacy and optimal digestive capacity are often difficult to achieve at the same time as numerous litters often require managerial practices that do not always put the “maturity” of the piglet first (e.g., early weaning or wide cross-fostering). Probiotics and prebiotics have attracted attention for this reason, and extensive studies have already been conducted to investigate their beneficial effects on animal performance and health [4,5,6]. Among these, yeasts and yeast-based products, in particular those obtained from the processing of *Saccharomyces cerevisiae,* have been used by several authors to improve the utilization and digestibility of nutrients, as well as to increase milk production in ruminants [7,8,9,10]. In the pig sector, various researchers have confirmed how a feed supplement based on these products can improve growth performance, milk production, nitrogen balance, nutrient digestion and reproductive performance [5,11,12,13]. Moreover, as the use of these products also had a good impact on piglets’ intestinal integrity, immunity and physical condition, this option can be used as a substitute for antibiotic growth promoters and, in general, as an alternative to antibiotics [14].

However, besides probiotics and prebiotics, the interest towards postbiotics has been increasing in recent years. Postbiotics are non-viable (inactivated) microorganisms in whole or part, or metabolites secreted by microorganisms that directly or indirectly improve the health status of hosts [15]. Their use is increasing because it is possible to become independent of the viability of the organisms and, therefore, they allow their use on a larger scale. Unlike living microorganisms, there are no risks of infection due to bacterial translocation from the intestinal lumen to the blood in susceptible and immunocompromised individuals [15]. Therefore, there is no possibility of acquiring and transferring antibiotic resistance genes. They could be added at a lower dosage than probiotics, and they are not inactivated during the feed manufacture, transport and storing [15]. Compared to probiotics, postbiotics offer more advantages; for instance, live probiotics may have trouble adhering to the gut mucosa because of the mucous layer that limits the direct contact between the gut mucosal layers and bacteria [14]. However, postbiotics can pass through the mucous layer quickly [14]. The use of postbiotics is highly recommended to increase productivity and decrease herd mortality through their effect on the balance restoration of the gut microbiota, immunomodulation or immunoprotection, pathogen protection and maintenance of the intestinal barrier integrity [14]. Specific studies on postbiotics obtained from *Saccharomyces cerevisiae* are published on sows [16], broilers [17], layer pullets [18], horses [19] and dogs [20]. However, a gap in the knowledge regarding the effects of postbiotics from inactivated and stabilized whole-cells of *Saccharomyces cerevisiae* is highlighted, as the majority of the studies used fermentation products. Moreover, minimal information is available regarding the passive effects that the administration of postbiotics to the sow during gestation can have on the litter up to the post-weaning phase.

The aim of this work is to determine the effects of a postbiotic obtained from inactivated and stabilized whole-cells of *Saccharomyces cerevisiae* administered during the gestation of the sow on the productive performance of the mother and litter during lactation. A specific focus was placed on lactation efficiency as a novel parameter to evaluate the capacity of the sow of well-using feed energy to wean more kilograms [21]. Moreover, piglets were followed in the post-weaning phase to investigate the possible passive effects of maternal supplementation on their health and antibiotic consumption. The research hypothesis tested was that the productive performances of the mother and litter during lactation were improved by the postbiotic administration during the sow’s gestation compared to the untreated control group. Moreover, reduced antibiotic consumption and mortality in piglets pre- and post-weaning were expected.

## 2. Materials and Methods

The study was hosted in a commercial breeding farm (750 productive sows) negative to porcine reproductive and respiratory syndrome virus (PRRSV) infection, positive to Mycoplasma hyopneumoniae and positive to Porcine circovirus 2 (PCV2) but without evidence of clinical expression. Piglets were vaccinated for Mycoplasma hyopneumoniae, PCV2, PRRSV and *Escherichia coli* F4/F18. The trial involved 183 sows (first and second parity, Danish Genetics^®®^), divided into two experimental groups four days after breeding, at the time of their group housing in the gestation facilities: (1) sows fed with basal gestation feed, daily supplemented with 50 mL of a postbiotic obtained from inactivated and stabilized whole-cells of *Saccharomyces cerevisiae* (Eubriotic Sow^®®^, Dox-al Italia, Sulbiate MB, Italy) from the fifth day of gestation until day 113 (treated group, *n* = 90); (2) sows fed with the same basal gestation feed, without any supplement (control group, *n* = 93). The basal diet is shown in Table 1. The whole-cells of *Saccharomyces cerevisiae* were obtained from a yeast culture and the inactivation process consisted of blocking the biological activity in the reproductive multiplication phase through a patented physical (heating) treatment, preserving the morphological aspect and cellular heritage of the living cell. The treatment ensured that the inhibition of the biological activity was maintained in the long-term, and, at the same time, the original integrity of the cell was preserved.

The day of recruitment (four days after service), the sows were moved from the insemination room to four identical group-pens (two group-pens for the treated group and two for the control group), divided into two identical rooms containing both the experimental groups, where they were housed until day 113 of gestation. Each group-pen individually fed each sow through an electronic feeding station, which dispensed the exact amount of feed to each sow after she was identified by means of a transponder in her ear tag. In the treated group, the supplemental product was stored in a closed compartment inside the electronic feeding station and dispensed via an automatic pump settled to deliver 50 mL once a day to each sow that poured it directly onto the meal in the feed bowl, at the time of individual feed dispensing. A short video showing this process is available in the Supplementary Materials (Video S1). The automated delivery of the postbiotic was studied to be easily compatible with the industrial size of the farm that hosted the study, reducing the need for labor by personnel. The housing of the animals observed the EU legal stocking density, and 0.2 kg/sow of chopped straw was daily and automatically distributed on the floor as environmental enrichment for the animals. On day 113 of gestation, the sows were moved to the individual farrowing pens, with a floor area of 7.2 m^2^ (of which 1.3 m^2^ was the nest reserved for piglets), where they naturally farrowed 2–3 days later. The sows were temporarily confined in cages only during the first three days after farrowing, after which they were allowed to freely move around the pen until weaning. Piglets were weaned at an average age of 28 days.

Daily ingestion of the sows was individually recorded during both gestation and lactation. At farrowing, the number of live born piglets and the total litter weight were recorded. Cross-fostering was drastically reduced to the minimum necessary for the survival of the piglets and was allowed only within the same experimental group. Furthermore, on the day of farrowing (0–3 h after the birth of the first piglet), the colostrum quality was assessed for each sow using an optical Brix refractometer (HHTEC, Heidelgerb, Germany) which estimated the IgG concentration directly on farm [22]. The Brix refractometer is used to measure % sucrose in liquids, and when used in non-sucrose-containing liquids approximates the percentage of total solids [23]. A 0.3 mL of freshly drawn colostrum sample was used for the on-farm measurement shortly after collection.

During lactation, piglets’ mortality and the consumption of antibiotics were recorded. A fecal score was also collected three times on litters (at days 3, 10 and 23 of lactation) using a 3-point scale (0 = absence of diarrhea; 1 = presence of at least one pasty discharge; 2 = presence of at least one liquid discharge). A bacteriological investigation on piglets’ feces was performed in both the experimental groups when the first clinical sign of enteric disease appeared. At weaning, the litters were weighed again in order to calculate their weight gain. A short video showing the breeding facilities of the experimental study is available in the Supplementary Materials (Video S1).

Weaned piglets were moved to two different weaning sites, historically affected by enterotoxigenic *Escherichia coli* (ETEC) clinical signs: weaning (1) housed 883 piglets in 4 rooms with 8 pens each (two rooms for the treated group = 499 piglets; two rooms for the control group = 384) with slatted floor, covered nests with heating floors, ad libitum dry feed (Table 2) and straw in racks as environmental enrichment; weaning (2) 1119 piglets were housed in 8 pens in the same barn (four pens for the treated group = 560; four pens for the control group = 559) with solid floor, ad libitum dry feed (Table 2) and wooden logs as environmental enrichment. As the study did not change how the farm practices/management were conducted, piglets were allocated by treatment in the pens disassembling the litter of origin and balancing the groups by size as visually assessed by the farm’s technicians.

In weaning 1, the animals were monitored for 57 days. During this period, the animals were weighted by pen two times (day 1 and day 57), and antibiotic consumption by pen and mortality were monitored. In weaning 2, the animals were monitored for 14 days, during which the feed intake by group, antibiotic consumption by pen and mortality were collected. In both the weaning sites, a bacteriological investigation on feces was performed at the first clinical sign of enteric disease.

**Figure 1:**
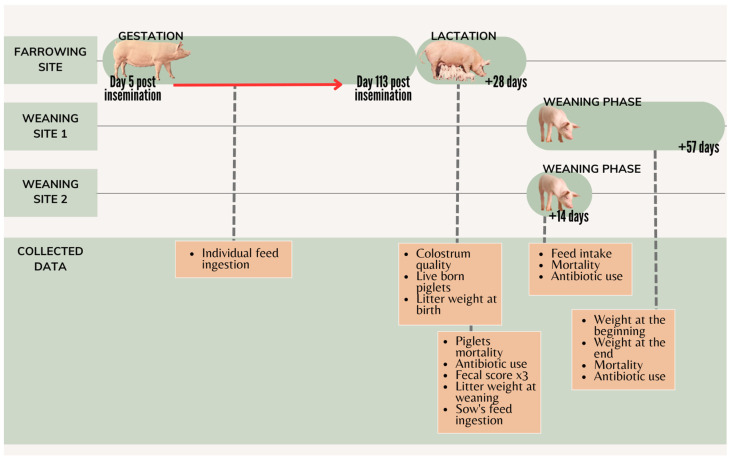
Timeline of the study. The red line indicates the period of the postbiotic administration to the treated group. Timepoints of the reproductive data collected during the next reproductive cycle of the sows are not shown.

Reproductive data from each sow were also collected after the next reproductive cycle. The timeline of the study is represented in Figure 1.

### Statistical Analysis

Statistical analysis was performed by XLSTAT 2022.2.1 (Addinson, TX, USA, 2022). Distribution of the data was tested using the Shapiro–Wilk test; considering that some parameters were not normally distributed (live born piglets/sow; average piglet’s birth weight; weaned piglets/sow; litter weight at weaning), the Mann–Whitney test was used for further analysis. Only mortality, expressed a frequency, was evaluated with the chi-squared test with the Yates correction. Statistical significance was set at *p*-values (*p*) < 0.05, while values lower than 0.10 were considered a trend. During lactation, the experimental unit was the sow with/or her litter, whether it was the pen during the weaning phase in both the weaning sites. Only for mortality, the experimental unit was the piglet both during lactation and the weaning phase.

Lactation efficiency was obtained using a modified version of the formula suggested by Bergsma et al. [21]:

Lactation efficiency (%) = output × 100/input(1)

where the output was the weight gain (kg) of the litter during lactation, and the input was the feed intake of the sow during the same period.

The defined daily dose/population correction unit (DDDvet/PCU) method proposed by EMA [24] was adopted to quantify the overall antimicrobial use per experimental group. The EMA provides standardized daily dosages for each active substance; the PCU was obtained by multiplying the total number of animals per group by a standard live weight at treatments of 4 kg for suckling piglets and 12 kg for the weaners.

DDDvet/PCU = total administered active substance (mg)/defined daily 
dosage (mg/kg/d) × *n* animals × expected weight at treatment (kg) (2)

For the data relating to the feed intake in the weaning site 2, as it was available only by group, a simple descriptive statistic was carried out and shown in the results.

## 3. Results

Feed consumption during gestation of sows belonging to the two experimental groups did not show significant differences. The ingestion for both groups was very close to the standard curve established for this production phase (expected consumption: 195.19 kg/sow; observed consumption: 195.23 ± 73.3 kg/sow, i.e., +0.06%; *p* > 0.05). However, the sows in the treated group ingested a lower amount of feed during their stay in the farrowing room (166.4 ± 35.3 vs. 181.3 ± 34.9 kg/sow; *p* = 0.019). The total number of piglets born from the 90 sows in the treated group was 1373; the number was 1443 piglets for the 93 sows in the control group. Among the performances of the lactating litter, only the piglets’ mortality showed a statistical difference between the experimental groups, with a lower percentage in the treated group (25.1 ± 16.7 vs. 28.8 ± 14.4%; *p* = 0.048; Table 3). A statistical tendency in favor of the treated group was also shown concerning the average weight of the piglets at birth (1.39 ± 0.3 vs. 1.32 ± 0.4 kg; *p* = 0.087; Table 3). Moreover, lactation efficiency improved by +5.9% in the treated group compared to the control one (41.3 ± 11.4 vs. 35.4 ± 11.6%; *p* = 0.011; Table 3). In both the experimental groups, bacteriological investigation on piglets’ feces identified the presence of ETEC. Antibiotics were administered to piglets only by injection for individual treatment of clinical signs. No metaphylaxis by mass medication was needed.

The piglets entering weaning 1 weighed 8.32 ± 2.41 and 7.81 ± 2.18 kg, respectively, for the control group and the treated group (*p* > 0.05) while, after 57 days, their average weight was 41.0 ± 7.2 and 43.1 ± 7.5 kg (*p* > 0.05). During this period, the treated group consumed less antibiotics than the control group (0.72 ± 0.25 vs. 1.22 ± 0.30 DDDvet/PCU; *p* = 0.047) and showed a statistical tendency for a mortality reduced by −42.3% (1.2 vs. 2.08%; *p* = 0.092).

In weaning 2, the piglets in the treated group daily consumed 221 gr/pig during their first 14 days of staying in the site, against 208 gr/pig in the control group (+6.2%). Mortality was 0.71 vs. 1.61%, respectively (reduced by −55.9% in the treated group; *p* = 0.10). The antibiotic consumption was 2.35 vs. 2.12 DDDvet/PCU (*p* > 0.05).

In both the weaning sites, bacteriological investigation on feces identified the presence of ETEC. Antibiotics were administered to piglets only by injection for individual treatment of clinical signs. No metaphylaxis by mass medication was needed during the study.

Reproductive parameters of the sows the next reproductive cycle are reported in Table 4. The total number of piglets born from the 90 sows in the treated group at the subsequent farrowing was 1584; the number was 1702 piglets for the 93 sows in the control group. No statistical differences were shown.

## 4. Discussion

Some characteristics of yeast-based products have already been highlighted by several studies in the past, which confirmed their ability to promote growth and potential properties to reduce the use of antibiotics [5,25,26,27] in pigs. In the present study, a postbiotic obtained from inactivated and stabilized whole-cells of *Saccharomyces cerevisiae* from yeast culture was used. Among the 500 different yeast species, *Saccharomyces cerevisiae* is the most commonly used in the feed industry as it received the Qualified Presumption of Safety status from the European Feed Safety Authority (EFSA). Several yeast-derivatives are available for livestock, both in the probiotic (e.g., viable yeasts) and prebiotic form (e.g., yeast-cell components). Besides them, the relatively new concept of postbiotic seems to be promising for the physiological benefits to the host. For this study, the selected postbiotic was produced by a treatment that ensured that the inhibition of the biological activity was maintained in the long term, and, at the same time, the original integrity of the cell was preserved. The process had been developed with the intention of maintaining all the beneficial factors of the viable cell, without the risk of creating antagonism in the organism due to bacterial translocation from the intestinal lumen to the blood in susceptible and immunocompromised individuals, increasing the ease of transport and storing, and the stability during the feed manufacture [15]. Viable cells from yeast culture, as reviewed by Perricone et al. [28], contain a rich variety of biologically active substances, including proteins, small peptides, oligosaccharides, vitamins, minerals, enzymes and numerous ‘unknown growth factors’, which can all exert beneficial nutritional and health effects on animals.

Some authors have attributed the ability of yeast-based products to provide reproductive benefits due to the predisposition towards a higher feed intake [27,29], increasing the average daily intake [5,27] and improving the feed efficiency of pigs [5]. Studies conducted using fermentation products obtained from *Saccharomyces cerevisiae* and administered to sows during gestation and lactation have shown that the benefit is the weight gain of the litter during lactation [12,13,16] and the improved immunological status of both the mother and the litter [30]. The present study failed in finding those latter differences between the two experimental groups, while there was a reduced mortality of suckling piglets and a tendency to have a higher average weight at birth in the treated group. One of the most logical hypotheses regarding the reduced mortality during lactation, followed by the same tendency in both the weaning sites, would be that the result is the consequence of an improved immunological status of the animals. However, the colostrum evaluation did not show any statistical difference between the experimental groups. Therefore, the authors suppose that the treatment might be helpful in the host defense towards the challenging conditions related to the ETEC infection in the piglets. This might also explain the reduced antibiotic use in weaning site 1 (probably the observation of the piglets in weaning site 2 for only 14 days was too short to show any difference). Several authors fully supported this hypothesis. During the development of ETEC infection, attachment to the intestinal mucosa is the first step in the pathogenesis of this pathogen, in particular, after weaning. For example, Daudelin et al. [31] and Kiarie et al. [32] found a reduced ETEC attachment to the ileal mucosa in animals treated with a *Saccharomyces cerevisae*-based product in comparison with an antibiotic-treated or untreated animals, respectively. Lessard et al. [33] found that this yeast may have the potential to modulate the local establishment of lymphocyte populations and IgA secretion in the gut and to reduce bacterial translocation to the mesenteric lymph node after ETEC infection. Shen et al. [27] also reported that the cytokine IFN-γ was increased in the intestinal mucosa following dietary yeast supplementation. Efficient phagocytosis of bacteria by macrophages activated by this cytokine can prevent the migration of pathogenic bacteria across the mucosa [34], thereby also reducing neutrophil and white blood cell counts. Even if, in the present study, the postbiotic was administered only to the mother, some authors suggest that the modulation of the maternal gut microbiota after a postbiotic administration might be later transferred to the offspring. For example, Xia et al. [30] found a reduced diarrhea incidence in piglets weaned from mothers previously fed with *Saccharomyces cerevisae.* Similar results were also reported by Betancur et al. [35] in lactating piglets. Indeed, Wang et al. [36] showed that to feed lactating sows with a supplement of yeasts resulted in the modification of the intestinal microbiota, associated with a decrease in the frequency of constipation in sows and diarrhea in piglets. In fact, piglets’ intestinal microbiota is highly modulated from the mother’s intestinal microbiota and environment. A previous study showed that oral administration of fecal microbiota obtained from healthy pigs to piglets suffering from diarrhea reduced the incidence of diarrhea [37]. This treatment boosted the immunity of the piglets and reduced stress-induced diarrhea. Considering that few field studies investigated the passive effects of yeasts on weaned piglets when administered only to the mother, further studies would be beneficial. Surely, a limitation of the present study is the absence of immunological and histopathological investigations in the weaning sites to confirm this hypothesis, as well as the lack of measurement regarding the numbers of fecal ETEC shedding. However, Brix investigation on the colostrum seems to suggest a generalized low level of colostrum quality that might be one of the causes of the historically reported enteric clinical signs in both the weaning sites. In fact, Hasan et al. [22] found that a Brix result between 20 and 24% in sows is related to an IgG estimation level between poor and adequate (borderline). The quality of the colostrum produced by the sow can be influenced by sow and litter characteristics, endocrine status, nutrition, environmental factors or a combination of these factors [38]. Quesnel [39] and Hasan et al. [22] observed that IgG concentrations in colostrum at parturition are not affected by parity, but the former reported that IgG concentrations in sows exceeding the fifth parity were greater than for first parity sows at 24 h postpartum. As the present study involved only first and second parity sows, the borderline colostrum quality might be related also to this factor. Moreover, it should be said that the piglets’ mortality recorded in this study during lactation in both the experimental groups is higher than those reported in the literature for hyperprolific sows (Baxter et al. [40] reported a lactational mortality close to 15%), but it might be related to the temporary crating of the sows in the farrowing pen. In fact, free lactation is reported as one of the major causes of piglets’ mortality due to crushing [41,42]. In the present study, lactation efficiency was improved in the group of treated sows. This parameter was first considered by Bergsma et al. [21], which describes the dynamics of body composition of sows and piglets during lactation, and introduces the new concept called “lactation efficiency”. Indeed, a parameter which would be able to evaluate the capacity of the sow of well-using feed energy to wean more kilograms is increasingly needed in modern pig production. Sow productivity has grown enormously in recent years, especially in the last decade, and genetic selection has led to larger litters, making relative the mere importance of other older parameters (e.g., number of weaned piglets/sow/year) if not also related to the weaning weight. In 2013, ‘very large’ litters (i.e., 21 + total piglets per sow) were considered relatively rare [43]. However, average data published since then from Denmark, arguably the most successful breeders of hyperprolific sow lines, show that total piglets born has continued to rise, with an extra 2.4 piglets born per litter since 2011 to 2018 [40]. It is normal to imagine how, to support such large litters, it is important to keep the sows in good physical condition. Feed intake of lactating sows is often not sufficient to sustain the milk production required for these litters [44,45]. Unfortunately, if the increased energy demand cannot be met by increased feed intake, sows mobilize energy from their own body reserves. This is not a problem if the mobilization is limited, while it becomes problematic if it is excessive, also resulting in fertility problems in the following reproductive cycle [46,47]. Better feed efficiency during lactation could be a solution: more milk produced with the same amount of feed ingested and the same mobilization of body reserves. The results obtained from the present study confirm what had already been suggested in 2009 by Bergsma et al. [21]: differences in lactation efficiency between different sows exist and can be influenced. The sows in the treatment group improved their lactation efficiency by more than +6%, without a reduced production in the next reproductive cycle. This is why the absence of differences in reproductive performances in the subsequent cycle should be considered a reached goal of the study. Stakeholders are accustomed to considering the feed conversion ratio only in the pig growth phase where, making an example of a 25 kg animal, 33% of the cost of the piglet is related to the feed [48], and more for heavier animals. The increase in feed efficiency during lactation is equally significant, and a 10% increase reduces the amount of feed required per sow/year by around 40 kg (calculation assumed by Bergsma et al. [21]). In the present study, a similar economy was observed, with around 15 kg per sow/farrowing saved.

Comparing the results and those obtained in other studies, a critical analysis of the duration of administration of the postbiotic should be taken into account. In fact, differently from the present study, other authors that reported positive results on the weight gain of the litter during lactation [12,13,16] also administered *Saccharomyces cerevisae* during lactation. Further studies on the best duration of feed supplementation would be useful, maybe also considering the hypothesis of a continuous treatment of sows and piglets after weaning.

A special consideration of the possible importance of future yeast use in livestock feed is linked to the so-called “cellular agriculture”. This relatively new term, coined in 2015 [49], means production of agricultural goods with animal or plant cell cultures or microbes or yeasts instead of using animals or crop plants. The rationale of cellular agriculture relies in the limited possibility to expand traditional agriculture. It has been supposed that crop production needs to increase by +100% between 2005 and 2050 to meet the food demand by the growing human population [50]. Unfortunately, the arable lands are limited and, actually, they are continuously lost and associated with environmental exploitation [51]. Microbial protein produced for food or feed is commonly referred to as a single cell protein; a wide variety of bacteria, fungi, yeasts, and microalgae has been studied as the sources of a single cell protein. Depending on the species and growth conditions, microbial or microalgal mass contains high concentrations of proteins ranging from 30 to 80% along with the other two basic macronutrients, lipids and carbohydrates [52]. Even though production of single cell proteins for feed is linked to animal farming and thereby again to agriculture, the utilization of non-edible raw materials still reduces arable land use and increases resource efficiency. *Saccharomyces cerevisiae* is listed among the heterotrophic microbes that might be considered a source of single cell proteins, on the condition that in these production processes, the microbes are not cultivated with starch-derived glucose as the substrate, avoiding the production ultimately dependent on agriculture [53]. The role of inactivated and stabilized whole-cells of *Saccharomyces cerevisiae* towards this concept should be investigated, supported by these findings related to the better feed efficiency observed in the treated group.

## 5. Conclusions

The results of this study suggest that dietary supplementation of a postbiotic obtained from inactivated and stabilized whole-cells of *Saccharomyces cerevisiae* from yeast culture during the gestation of sows can potentially improve lactation efficiency, mortality of the piglets and antibiotic use during the weaning phase. The findings are particularly important, especially in the era of modern pig farming, considering the increase in the cost of raw materials, the demand for increasingly performing animals and, at the same time, the need to decrease the consumption of antibiotics.

## Figures and Tables

**Table 1:** Ingredient compositions and nutrient levels of gestation and lactation diets (%, as-fed basis). CP: crude protein.

Item	Gestation 1 (0 to 90 Days)	Gestation 2 (90 to 113 Days)	Lactation
Ingredients			
Barley	34.0	29.4	23.7
Corn	20.0	17.5	25.9
Soft wheat bran	14.0	14.3	20.0
Wheat middling	10.0	7.0	8.0
Beet pulp	7.0	15.0	3.0
Soybean meal, 48% CP	6.5	7.8	12.8
Soft wheat	5.0	6.0	0.33
Calcium carbonate	1.2	0.5	1.0
Soybean oil	1.2	1.1	2.3
Fish meal	-	-	2.0
Vitamin and mineral premix *	0.4	0.9	0.52
Mono-dicalcium phosphate	0.35	-	0.37
Sodium chloride	0.3	0.5	-
D1-Methionine, 98%	0.08	0.09	0.04
L-Tryptophan, 98%	0.04	0.04	0.01
Total	100	100	100
Nutrient levels			
Dry matter	87.2	87.4	87.6
Crude protein	13.4	13.6	16.1
Crude fat	3.4	3.3	4.6
Fiber	5.7	7.0	3.7
Metabolizable energy, kcal/kg	2996.7	2999.2	3241.9
Valine	0.74	0.76	0.89
Lysine	0.57	0.63	0.77
Methionine + cysteine	0.52	0.53	0.57
Threonine	0.47	0.49	0.59
Tryptophan	0.20	0.20	0.20

* Vitamins and trace minerals per gram of premix: Cu, 6 mg; I, 0.5 mg; Mn, 24.9 mg; Se, 0.09 mg; Fe, 52.9 mg; Zn, 33.2 mg; vitamin A, 6500 IU; vitamin D3, 1250 IU; vitamin E, 2.3 mg; vitamin K3, 0.2 mg; vitamin B1, 0.1 mg; vitamin B2, 0.3 mg; vitamin B6, 0.12 mg; vitamin B12, 0.002 mg; pantothenic acid, 1 mg; vitamin H, 1.6 mg; vitamin PP, 1.6 mg.

**Table 2:** Ingredient compositions and nutrient levels of the feed administered in both the weaning sites (%, as-fed basis). CP: crude protein; BW: body weight.

Item	Weaning 1 (6 to 12 kg of BW)	Weaning 2 (12 to 23 kg of BW)	Weaning 3 (23 to 30 kg of BW)
Administered in weaning site 1	Yes	Yes	Yes
Administered in weaning site 2	Yes	No	No
Ingredients			
Wheat, 11% CP	25.0	18.0	13.0
Soft wheat bran	-	-	11.6
Barley, 11% CP	20.0	22.9	15.7
Milkiwean^®®^ mix ^1^	15.0	7.5	-
Bakery meal	7.25	10.0	-
Wheat bran, 15% CP	5.0	6.0	-
Whey, 20% added fat	5.0	2.5	-
Corn, 8% PG	5.0	12.0	37.0
Soybean meal, 44% CP	5.0	10.5	-
Soybean meal, 48% CP	-	-	15.4
Milkiwean Vital^®® 1^	5.0	5.0	-
Soybean oil	3.0	2.0	1.6
Pea, 22% CP	-	-	2.0
Fish meal	2.5	2.5	-
Plasma proteins	1.5	-	-
Benzoic acid	0.5	0.3	-
Selacid^®® 1^	0.25	0.15	0.3
Calcium carbonate	-	0.5	1.4
Mono-dicalcium phosphate	-	-	0.7
Sodium chloride	-	-	0.5
Vitamin and mineral premix	-	-	0.7
Total	100	100	100
Nutrient levels			
Dry matter	90.2	89.5	87.5
Crude protein	17.3	17.2	16.4
Crude fat	7.2	6.0	4.0
Fiber	3.3	3.7	3.2
Metabolizable energy, kcal/kg	3392.0	3312.0	3201.4
Lysine	1.2	1.3	1.0
Threonine	0.90	0.86	0.70
Methionine + cysteine	0.86	0.83	0.65
Tryptophan	0.27	0.25	0.20

^1^ Trouw nutrition Italia, Nutreco group, Mozzecane, VR, Italy.

**Table 3:** Productive data from the two experimental groups during the lactation phase.

Parameter	Treated Group (*n* = 90 Sows; 1373 Piglets)		Control Group (*n* = 93 Sows; 1443 Piglets)		*p*-Value
	Mean	Standard Deviation	Mean	Standard Deviation	
Live born piglets/sow (*n*)	15.3	1.4	15.5	1.3	0.208
Litter weight at birth (kg)	21.0	4.5	20.2	4.8	0.210
Average piglet’s birth weight (kg)	1.39	0.32	1.32	0.36	0.089
Weaned piglets/sow (*n*)	11.4	2.5	11.0	2.3	0.429
Litter weight at weaning (kg)	74.9	20.5	71.9	19.5	0.398
Average piglet’s weight at weaning (kg)	6.63	1.29	6.61	1.50	0.987
Litter’s fecal score (check 1, day 3)	1.35	0.56	1.27	0.49	0.511
Litter’s fecal score (check 2, day 10)	1.10	0.42	1.02	0.15	0.389
Litter’s fecal score (check 3, day 23)	1.06	0.43	1.09	0.46	0.258
Piglet’s average weight gain (kg)	5.24	1.24	5.29	1.46	0.746
Litter’s average weight gain (kg)	53.9	20.0	51.7	20.0	0.529
Brix * (%)	20.28	5.77	20.91	4.93	0.510
Lactation efficiency (%)	41.3	11.4	35.4	11.6	0.011
Piglets’ mortality (%)	25.1	16.7	28.8	14.4	0.048
Piglets’ antibiotic consumption (DDDvet/PCU)	3.32	1.78	3.29	1.95	0.487

* Colostral Brix reading: it approximates the percentage of total solids in colostrum, estimating the IgG concentration.

**Table 4:** Reproductive parameters from the two experimental groups during the next reproductive cycle.

Parameter	Treated Group (*n* = 90 Sows; 1584 Piglets)		Control Group (*n* = 93 Sows; 1702 Piglets)		*p*-Value
	Mean	Standard Deviation	Mean	Standard Deviation	
Live born piglets/sow (*n*)	17.6	3.9	18.3	3.8	0.232
Total born piglets/sow (*n*)	18.5	4.1	19.5	4.0	0.210
Stillborn piglets/sow (%)	6.2	10.7	8.3	12.2	0.108
Mummified piglets/sow (*n*)	2.9	6.0	3.6	10.9	0.911
Piglets’ mortality (%)	24.2	19.5	24.9	16.9	0.362
Weaned piglets (*n*)	11.1	2.9	10.8	3.0	0.355

## Data Availability

Not applicable.

## Supplementary Materials

The following supporting information can be downloaded at: https://www.mdpi.com/article/10.3390/vetsci10090576/s1, Video S1: The breeding facilities of the experimental study.
